# Evaluation of One Hundred Pediatric Muscle Biopsies During A 2-Year Period in Mofid Children And Toos Hospitals

**Published:** 2013

**Authors:** Yalda NILIPOR, Fakhredin SHARIATMADARI, Fatemeh ABDOLLAH GORJI, Mohsen ROUZROKH, Mohamad GHOFRANI, Parvaneh KARIMZADEH, Moahammad Mehdi TAGHDIRI, Hosein DELAVARKASMAEI, Farzad AHMADABADI, Mohammad Kazem BAKHSHANDEH BALI, Hamid NEMATI, Sasan SAKET, Narges JAFARI, Omid YAGHINI, Seyed Hasan TONEKABONI

**Affiliations:** 1 Neuropathologist, Mofid Children Hospital and Myopathology Lab of Toos Hospital, Tehran, Iran; 2 Fellowship of Pediatric Neurology, Pediatric Neurology Research Center, Shahid Beheshti University of Medical Sciences, Tehran, Iran; 3 Msc, Health Information Management Clinical Research Development Center, Mofid Children Hospital, Shahid Beheshti University of Medical Sciences, Tehran, Iran; 4 Assistant Professor of Pediatric Surgery, Shahid Behehshti University of Medical Sciences, Tehran, Iran; 5 Professor of Pediatric Neurology, Pediatric Research Center, Shahid Beheshti University of Medical Sciences, Tehran, Iran; 6 Professor of Pediatric Neurology, Pediatric Neurology Department, Mofid Children Hospital, Faculty of Medicine, Shahid Beheshti University of Medical Sciences, Tehran, Iran; 7 Associate Professor of Pediatric Neurology, Pediatric Neurology Research Center, Shahid Beheshti University of Medical Sciences, Tehran, Iran; 8 Assistant Professor of Neurology, Shahid Beheshti University of Medical Sciences, Tehran, Iran; 9 Assistant Professor of Pediatric Neurology, Pediatric Department, Isfahan University of Medical Sciencs, Isfahan, Iran

**Keywords:** Muscle biopsy, Congenital myopathy, Muscular dystrophy, Merosin, EMG

## Abstract

**Objective:**

Muscle biopsy is a very important diagnostic test in the investigation of a child with suspected neuromuscular disorder. The goal of this study was to review and evaluate pediatric muscle biopsies during a 2-year period with focus on histopathology diagnosis and correlations with other paraclinic studies.

**Materials & Methods:**

We investigated 100 muscle biopsies belonging to patients with clinical impression of neuromuscular disorder. These patients have been visited consecutively by pediatric neurologists during 2010 to 2012. Samples were investigated by standard enzyme histochemical and immunohistochemical techniques.

**Result:**

Sixty-nine (69%) males and 39 (39%) females with a mean age of 5.7 years were evaluated. Major pathologic diagnoses were Muscular dystrophy (48 cases), Neurogenic atrophy (18 cases), nonspecific myopathic atrophy (12cases), congenital myopathy (6 cases), storage myopathies (4 cases) and in 6 cases there was no specific histochemical pathologic finding. EMG was abnormal in 79 cases. Degree of correlation between EMG and biopsy result was significant in children ≥ 2 years of age.

**Conclusion:**

This study confirms the high diagnostic yields of muscle biopsy especially only if standard and new techniques such as enzyme study and immunohistochemistry are implemented. Also, we report 11 cases of Merosin negative congenital muscular dystrophy. This is the largest documented case series of Merosin deficient congenital muscular dystrophy reported from Iran.

## Introduction

Disease of the motor unit is common in children. These neuromuscular diseases may be genetically determined, congenital or acquired, acute or chronic, and progressive or static (1).

Neuromuscular disorders have limited clinical expression with considerable overlap of symptoms and signs (2).

Because of availability of new specific therapies for many diseases and for genetic and prognostic implications, precise diagnosis is very important (1). Definitive diagnosis depends on the proper applications of laboratory techniques (2). The procedures used most, are serum myogenic enzymes, electro myography (EMG), and muscle biopsy (2).

Muscle biopsy has become the most essential diagnostic procedure in evaluating a child with neuromuscular disease.

To date, there is no comprehensive study about prevalence of muscular disorders among Iranian children. 

The present study was performed to evaluate actual status of neuromuscular disorders in our patients who were candidate for muscle biopsy.

## Materials & Methods

We have retrospectively reviewed the medical records concerning muscle biopsies belong to 100 children with clinical impression of neuromuscular disorder referred for muscle biopsy in Mofid and Toos hospitals from September 2010 to September 2012. 

A pediatric neurologist initially visited all 100 children aged between 4 months to 15 years old.

Based on medical history and physical examination, myogenic enzymes and EMG study they were candidate for muscle biopsy.

All data, including age, sex, EMG reports, myogenic enzymes, family history and consanguinity of parents were collected.

All muscle samples delivered immediately to myopathology lab in fresh state and were frozen in isopentane cooled in liquid nitrogen and a standard panel of histochemical stains (H&E, trichrome Gomori, Congo red, PAS, Oil red O, NADH-TR, SDH, Cox, Cox+SDH and ATPase x3) were performed for all the samples. Immunohistochemical study was performed for 43 patients composed of antibodies against Dystrophin (DYS1,2,3), alpha and gamma Sarcoglycans, Dysferlin, Merosin and also beta-Spectrin as a positive control.

The collected data were analyzed with statistical package for the social sciences (SPSS) soft ware (version 18), Also, chi-Square test or Fisher’s exact test were performed for categorical variables.

## Results

**Fig1:**
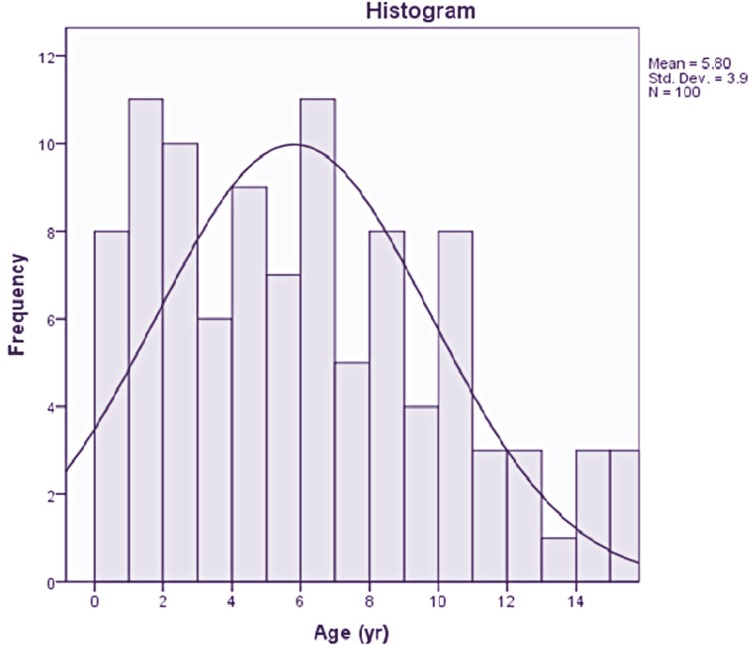
Distribution age histogram

In this study100 patients (61% males and 39% females) were included. The patients at the time of muscle biopsy aged between 4 months to 15 years (Figure1).The results indicated that only 20 cases had family history of muscle diseases (20% positive family history), fifty-eight patients were result of a consanguinity marriage.

The diagnostic results were as follow: muscular dystrophy was reported in 48 cases, out of which 10 were Duchenne muscular dystrophy, 13 were reported as Sarcoglycanopathy, among them 2 cases were suggested as alpha, 5 as gamma and 6 cases were suggested as beta sarcoglycanopathy. One of the dystrophic patients suffered from Dysferlinopathy and in 11 cases Merosin negative congenital muscular dystrophy was confirmed. IHC study was normal in 8 cases of muscular dystrophy and in 5 cases the data on IHC study is not available. 

Neurogenic atrophy was diagnosed in 18 cases, among them 14 cases were clinically or genetically confirmed as spinal muscular atrophy. 

Metabolic myopathies were diagnosed in 4 cases composed of 2 cases of juvenile Pompe and 2 cases of lipid storage myopathy. Congenital myopathy was reported in 6 cases out of which 4 were congenital myopathy with core lesions and 2 were Nemaline myopathy. One case of dermatomyositis was diagnosed. 

Selective type 2 fibers atrophy was seen in 5 cases and in 6 cases no specific histochemical pathologic finding was seen (Table 1).

Creatine Kinase was within abnormal limit in 66 cases and EMG was abnormal (myopathic or neurogenic patterns) in 79 cases.

**Fig 2:**
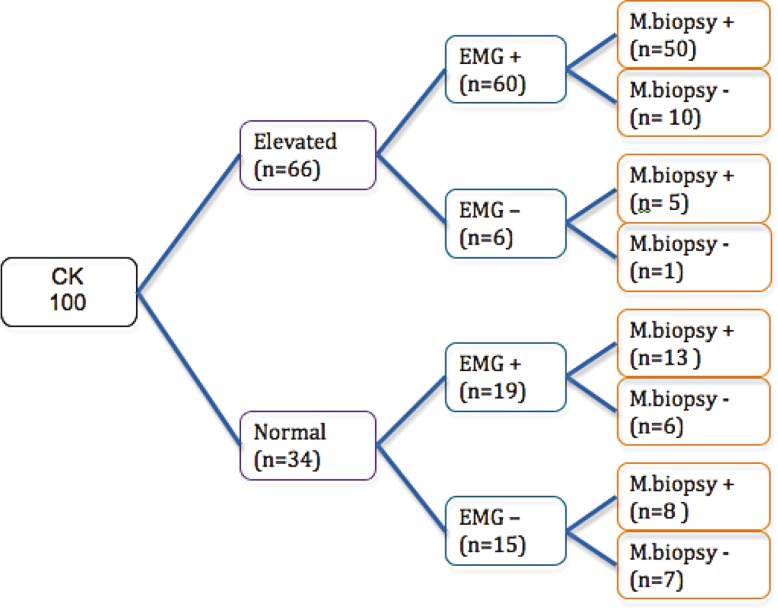
Correlation among CK and BIOPSY and EMG RESULTS, EMG+: myopathic or neurogenic patterns, EMG- : normal patterns, M.Biopsy+: any kinds of myopathic results in muscle biopsy,M.Biopsy - :Muscle biopsy with no specific pathologic finding

There is a correlation in 50 cases (50%) of patients between abnormal CK level, EMG and muscle biopsy result as any myopathic finding (Figure 2).

In spite of positive correlation between EMG and CK level in cases of myopathic results, biopsy results from 10 cases didn’t demonstrate myopathy (Figure 2).

Degree of correlation between biopsy and EMG in patients less than 2 years of age was 18/3%, while in children more than 2 years of age was (82%), the difference between the 2 groups is statistically significant (Pvalue=0.012) (Table 2).

## Discussion

In this study we do not present the frequency of neuromuscular disorders in Iranian children as we did not study 100 patients entering the neuromuscular clinic and we just studied 100 biopsy samples from patients who were candidates for muscle biopsy and this data is not representative of prevalence or frequency of muscle diseases in Iranian children.

Based on the present data muscular dystrophies were the most common type of neuromuscular disorders (48%) in our study like other studies (3, 4), but in one study that was taken in India; SMA was the most common disorders (5).

The main reason is patient selection method; as many Iranian neurologists order “molecular genetic test“ as the first-line work up in cases suspected of having SMA.

We found 11cases (11%) of Merosin negative congenital muscular dystrophy in our study, while the incidence of this disorder was approximately 10 times more than the study reported from India (5). We believe that this is not because of the higher prevalence of Merosin negative congenital muscular dystrophy in Iranian population but maybe it is due to our method of patient selection. Our study presents the largest documented case series of Merosin deficient congenital muscular dystrophy reported from Iran.

We collected 6 cases with no specific histopathologic finding. The paucity of no specific findings in our muscle biopsy specimens may be due to orthodox selection of candidates for muscle biopsy.

Degree of correlation between biopsy and EMG in patients < 2 years of age was 18/3%, while in children ≥ 2 years of age was 82%, the difference between the two groups is statistically significant (p value=0.012). These data show that EMG results are not reliable for children less than 2 years of age. This is in concordance with another study which shows EMG detection rate of myopathic motor unit potentials at a young age as low, improving in children over 2 years of age (6).

This study confirms the high diagnostic yields of muscle biopsy especially only if standard and new techniques such as enzymes study and immunohistochemistry are implemented.

**Table1:** Muscle Biopsy Results

	**Frequency**	**Percent**
Neurogenic atrophy	18	18.o
Sarcoglycanopathy	13	13.0
Nonspecific myopathic atrophy	12	12.0
Merosin Negative congenital muscular atrophy	11	11.0
Dystrophinopathy	10	10.0
Dystrophy with normal IHC	8	8.0
NO Specific histochemical pathologic finding	6	6.o
Dystrophy without IHC study	5	5.0
Selective type 2 fibers atrophy	5	5.o
Congenital myopathy with cores	4	4.o
Lipid storage myopathy	2	2.0
Glycogen storage myopathy	2	2.o
Nemaline Myaopathy	2	2.o
Dermatomyositis	1	1.o
Dysferlinopathy	1	1.o
Total	100	100

**Table 2:** Comparison of Muscle Biopsy with EMG based on Age Groups

**Age**	**EMG**		**Total**
	**Negative**	**Positive**	
2>	9 (50%)	15 (18.3%)	24 (24%)
2≤	9 (50%)	67 (81.7%)	76 (76%)
	18	82	100
